# Anticancer Targets and Signaling Pathways Activated by Britannin and Related Pseudoguaianolide Sesquiterpene Lactones

**DOI:** 10.3390/biomedicines9101325

**Published:** 2021-09-26

**Authors:** Christian Bailly

**Affiliations:** OncoWitan, Scientific Consulting Office, 59290 Lille, France; christian.bailly@oncowitan.com

**Keywords:** britannin, cancer, *Inula* species, NFκB, Nrf2, HIF-1α, PD-L1, sesquiterpene lactones

## Abstract

Sesquiterpene lactones (SLs) are abundant in plants and display a large spectrum of bioactivities. The compound britannin (BRT), found in different *Inula* species, is a pseudoguaianolide-type SL equipped with a typical and highly reactive α-methylene-γ-lactone moiety. The bioproperties of BRT and related pseudoguaianolide SLs, including helenalin, gaillardin, bigelovin and others, have been reviewed. Marked anticancer activities of BRT have been evidenced in vitro and in vivo with different tumor models. Three main mechanisms are implicated: (i) interference with the NFκB/ROS pathway, a mechanism common to many other SL monomers and dimers; (ii) blockade of the Keap1-Nrf2 pathway, with a covalent binding to a cysteine residue of Keap1 via the reactive α-methylene unit of BRT; (iii) a modulation of the c-Myc/HIF-1α signaling axis leading to a downregulation of the PD-1/PD-L1 immune checkpoint and activation of cytotoxic T lymphocytes. The non-specific reactivity of the α-methylene-γ-lactone moiety with the sulfhydryl groups of proteins is discussed. Options to reduce or abolish this reactivity have been proposed. Emphasis is placed on the capacity of BRT to modulate the tumor microenvironment and the immune-modulatory action of the natural product. The present review recapitulates the anticancer effects of BRT, some central concerns with SLs and discusses the implication of the PD1/PD-L1 checkpoint in its antitumor action.

## 1. Introduction

**Figure 1:**
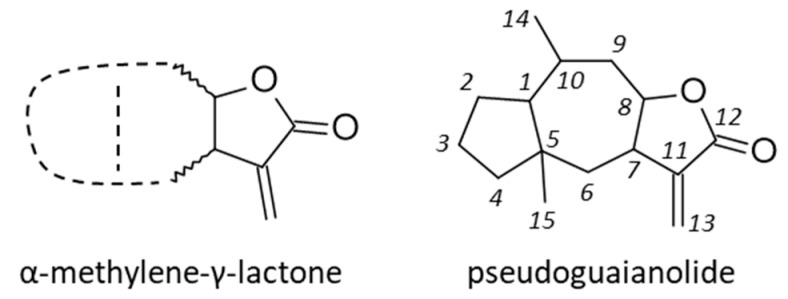
Structures of the α-methylene-γ-lactone unit and pseudoguaianolide core, present in britannin and related products. The numbering scheme is mentioned.

Sesquiterpene lactones (SLs) represent a large class of natural products found in many plant species. The compounds are characterized by the presence of an α-methylene-γ-lactone moiety appended to a mono- or bi-cyclic system (Figure 1). The nature of the structural backbone defines several sesquiterpene lactone subclasses bearing a germacranolide, heliangolide, eudesmanolide, xanthanolide, elemonolide, guaianolide or pseudo-guaianolide carbocylic skeleton [1]. They all contain a highly electrophilic α,β-unsaturated carbonyl moiety which can react easily with biological nucleophiles, such as thiol-containing molecules. They usually display prominent antioxidant and anti-inflammatory properties. Many natural products in this large family have been considered for the treatment of human diseases, including inflammatory diseases, metabolic syndrome, parasitic diseases and cancers [2,3,4].

**Figure 2:**
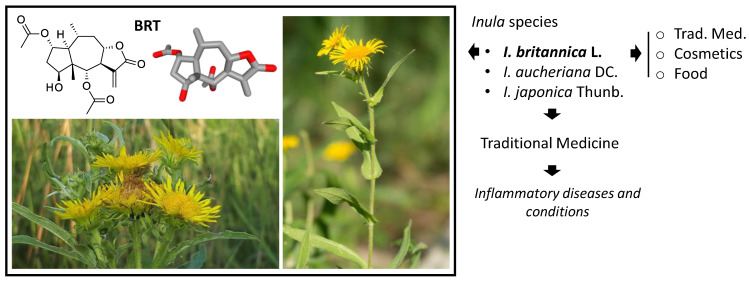
Chemical structure and conformation of britannin (BRT), isolated from the plant *Inula britannica* L. (photos accessible from https://identify.plantnet.org/fr/weurope/species/Inula%20britannica%20L./data, accessed on 25 September 2021). Each plant produces several yellow ray flowers, positioned on a long flower stalk. BRT can be isolated from other *Inula* species, such as *I. japonica*, *I. aucheriana*, and *I. oculus-christi*.

In this class of natural products, the subgroup of pseudo-guaianolides (or *pseudo-guaiacanes*) has been less investigated than other SLs. This subgroup includes the lead compound helenalin being potently active but causing allergic reactions [5]. The subclass also includes analogues such as mexicanin I, damsin and neoambrosin [6,7,8]. Another important member of the group is britannin (BRT in Figure 2), initially isolated from the plant *Inula britannica* L. [9]. These compounds have been characterized as inhibitors of the transcription factor NFκB, due to the targeting of a cysteine residue of the p65 subunit by the α-methylene-γ-butyrolactone moiety [10,11]. However, the mechanism of action at the origin of the anticancer properties of BRT is multifactorial, with several protein targets implicated or at least proposed. The present review provides a survey of the anticancer properties of BRT, with an analysis of the mechanism of action and targets for this interesting SL natural product. The goal is to dissect the signaling pathways implicated in the modulation of the tumor and its microenvironment, and to try to guide future drug design in this chemical series.

## 2. *Inula* Species Producing BRT and Their Medicinal Uses

The plant genus *Inula* (Asteraceae) comprises more than 100 species distributed worldwide (www.theplantlist.org, accessed on 25 September 2021). BRT was first isolated from *I. britannica* L. in 1968 [9,12,13], but later the product was found in other *Inula* species [14], such as I. aucheriana DC. [15] and I. japonica Thunb. [16,17]. *I. britannica* is an erect herb about 50–75 cm tall, with lance-shaped leaves. Each plant produces many yellow ray flowers, positioned on a long flower stalk (Figure 2). The plant is native to regions of Europe and Asia. It was introduced in North America at the beginning of the XXth century. BRT is one of the major natural products isolated from the medicinal plant I. japonica. This plant, designated British yellowhead (or meadow fleabane), is known as Xuanfuhua in traditional Chinese medicine (TCM) to treat sputum, and occasionally as a remedy for nausea, vomiting, hiccups, and flatulence [18,19]. A few years ago, an ethanolic extract of *I. japonica* was advanced to clinical trials in South Korea for the treatment of chronic bronchitis and asthma [20]. The anti-asthmatic activity of Inulae flos extracts is linked to the presence of BRT in the extract [21]. In Japan, *I. britannica* is used in Kampo medicine to treat nausea, hiccups, and excessive sputum [22]. In Iranian folk medicine, the plant is utilized in the treatment of arthritis and back pain [23].

Extracts of *I. britannica* display a range of pharmacological effects, including antinociceptive [23], antimicrobial [24,25], anticonvulsant [26], antiviral [27], antiaging [28] and hepatoprotective effects [29]. In most cases, these studies refer to aqueous and alcoholic (mostly methanolic) extracts, which essentially contain a panoply of flavonoids and terpenoids. Sesquiterpene lactones are usually present in these extracts, including but not limited to BRT [30,31,32,33]. In cases of water extraction, the extracts also include polysaccharides contributing to the bioactivity, notably as antidiabetic agents [34]. However, in most cases, the bioactivities are supported by sesquiterpenoid monomer and dimers, largely represented in these extracts. Inula sesquiterpenoids are extremely diversified, both structurally and functionally [13,17,35]. BRT is one of the many SLs isolated from Inula species.

Extracts of *I. britannica* flowers can be used in cosmetic products [36] to take advantage of the skin anti-inflammatory, antioxidant, antibacterial and anti-aging properties of the extract [37,38,39]. A methanol extract of the dried flower of *I. britannica* L. has been shown to suppress the expression of the tyrosinase enzyme implicated in melanin synthesis. Different sesquiterpenes, such as britannilactone, have been implicated in this anti-melanogenic activity [40]. The plant can be used to design depigmenting or skin-whitening products and to treat hyper-pigmentary disorders [41,42]. Alternatively, the use of an *I. britannica* flower petal extract fermented by a *Lactobacillus* species has been reported, leading to a product with an enhanced skin whitening activity due to increased tyrosinase activity [43]. The bioavailability of *I. britannica* can be significantly enhanced also upon fermentation with probiotic *Weissella cibaria* D30, so as to reduce the toxicity and increase the anti-inflammatory properties of the plant extract [44]. The applications of *I. britannica* extracts go beyond medicine and cosmetics, with uses in the food industry. A cheddar cheese fortified with an extract of *I. britannica* has been proposed. The plant extract served to increase the odor and taste of the cheese, while providing an antioxidant supplement [45].

## 3. Discovery, Structural Characterization and Synthesis of BRT

**Figure 3:**
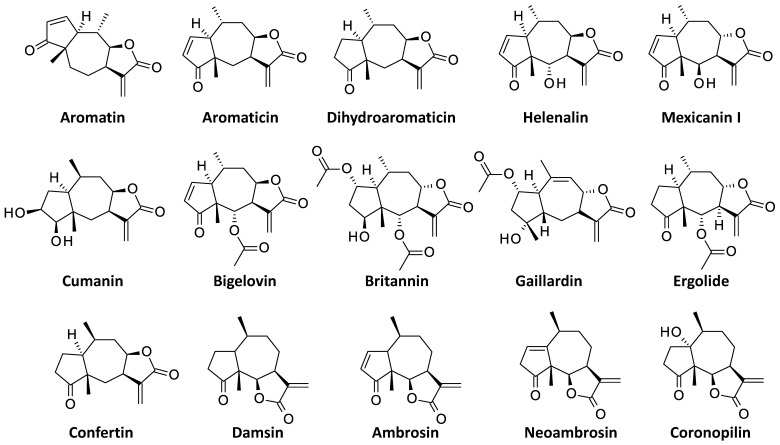
Structures of different pseudoguaianolide-type sesquiterpene lactones. The lactone ring can be *cis*- or *trans*-annelated to the seven-membered ring via the 7,8-positions (as for BRT) or via the 6,7-positions (as for damsin, (neo)ambrosin, and coronopilin). Pseudoguaianolides with a 15-methyl group on C-5 differ from pseudoguaianolides which have the methyl group on C-4.

As mentioned above, BRT was first isolated from a flowering sample of *I. britannica* collected in the Muyunmum desert (Jambyl region, southern Kazakhstan) in 1968 by Russian scientists [9]. The compound was identified as a sesquiterpene lactone and its structure was elucidated a few years later by the same authors [12]. BRT is a of pseudo-guaianolide, structurally similar to helenalin and mexicanin I. It is also structurally close to ergolide, bigelovin and 2,3-dihydroaromaticin, which are other pseudo-guaianolide-type sesquiterpene lactones found in *I. britannica* (Figure 3) [46,47,48]. The pseudoguaianolide core has been shown to adopt a twisted boat conformation in the crystal structure [49] but the tricyclic core of BRT itself appears relatively flat (Figure 2) [50].

The chemical synthesis of sesquiterpene lactones is feasible, although the multi-step processes are generally complex and difficult. Synthetic schemes have been reported for the germacranolide skeleton and specific SLs, such as parthenolide (from the medicinal plant *Tanacetum parthenium*). Synthesis has also been reported for the guaianolide-type SLs arglabin and thapsigargin [51] and for eremanthine [52,53]. The synthesis of pseudoguaianolide analogues has been described recently [54], and the synthesis of confertin and helenalin have been described a long time ago [55,56,57]. The complete synthesis of other pseudoguaianolides, such as aromatin, aromaticin, damsin, confertin and mexicanin I, has been presented [58,59], but to our knowledge, the total synthesis of BRT has not been reported. Only hemi-synthetic compounds have been described, such as functionalized thio-analogues bearing a methyl mercaptoacetate substituent at position 13 [60].

## 4. Anticancer Properties

The capacity of BRT to inhibit cancer cell proliferation has been demonstrated with cell lines derived from onco-hematological malignancies and solid tumors. The compound dose-dependently inhibits cell proliferation and triggered apoptosis of the breast cancer cell lines MCF-7 and MDA-MB-468 cells in vitro, through the activation of the mitochondrial apoptotic pathway. The intrinsic potency of the compound is not spectacular (IC_50_ = 9.6 and 6.8 μM, for MCF-7 and MDA-MB-468 cells, respectively) but BRT proved to be an efficient activator of reactive oxygen species (ROS) contributing to apoptosis [61]. In fact, another study indicated that the compound could reduce proliferation and trigger apoptosis of cancer cells of different origin with a roughly similar efficacy. The calculated IC_50_ values ranged from 2.3 to 5.9 μg/mL (6.3 to 16.1 μM) with MCF-7 and Madin–Darby bovine kidney (MDBK) cells, respectively, and intermediate values with other cell lines [15].

BRT reduces proliferation and triggers apoptosis of liver cancer cells, with the same efficacy as observed with breast cancer cells and with a comparable ROS-regulated mechanism (IC_50_ = 6.9 μM with HepG2 cells after 48 h of BRT treatment). Interestingly, the compound was found to induce both apoptosis and autophagy of HepG2 cells and, importantly, to reduce tumor growth in vivo. A daily intraperitoneal treatment of mice bearing HepG2 tumors with BRT at 7.5, 15 and 30 mg/kg reduced the tumor growth in a dose-dependent manner [62]. More recently, the antitumoral activity of BRT against hepatocellular carcinoma in vivo has been confirmed using BEL-7402 cells which are more sensitive to BRT compared to HepG2 cells. BRT inhibited the migration of the tumor cells, and bioluminescence imaging revealed that the compound markedly reduced the size of tumors in mice [63].

**Figure 4:**
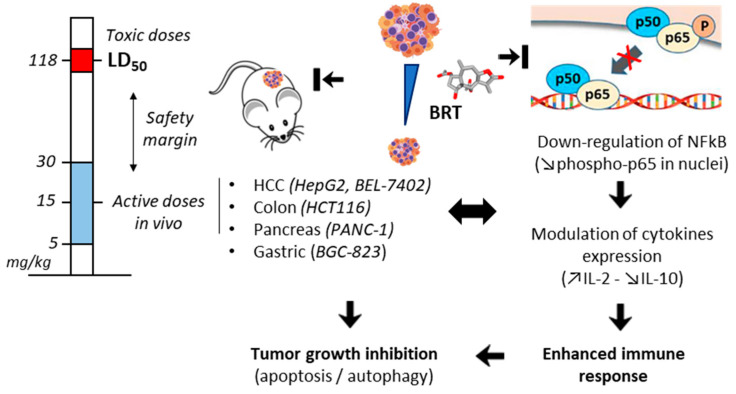
Anticancer activity of BRT in xenograft mice models of solid tumors. The compound was found to reduce tumor growth when administered (intraperitoneally) at the dose of 5–30 mg/kg (active doses), well inferior to the toxic dose (LD_50_) determined in an acute toxicity study (derived from [65]). The contribution of NFκB inhibition and immune regulation to the anticancer action of action of BRT is highlighted. Inhibition of p65 nuclear expression and up-regulation of interleukin-2 (IL-2) coupled to down-regulation of IL-10 expression have been evidenced in BGC-823 gastric tumors [66]. BRT-induced immune response plays a marked role in the antitumor action.

The pro-apoptotic functions of BRT have been evidenced with other types of cancers, including pancreatic cancer [64,65], gastric cancer [66] and colon cancer [67] (Figure 4). In each case, BRT demonstrated a robust capacity to reduce tumor cell proliferation and to induce apoptosis in vitro and to inhibit tumor growth in vivo. With HCT116 colon cancer cells, BRT revealed a prominent activity in vivo, completely abolishing tumor growth in mice when the compound was administered at the dose of 15 mg/kg every 3 days for about one month. A spectacular tumor growth inhibition, without apparent toxicity, was observed in mice [67]. The compound is well tolerated. In an acute toxicity test, the calculated low lethal dose 50% (LD_50_) value was 117.6 mg/kg, whereas a firm antitumor activity was observed upon repeated treatment with BRT at 5 and 10 mg/kg, in mice bearing PANC-1 pancreatic tumors [65]. In onco-hematology, the effect of BRT has been little investigated thus far. However, a recent study highlighted the capacity of the compound to reduce the proliferation of acute lymphoblastic leukemia (ALL)-derived MOLT-4 cells and to produce a synergistic effect with the tubulin-binding drug vincristine in vitro [68]. A similar effect has been observed with the related sesquiterpene lactones gaillardin and ergolide which also exert marked antileukemic effects in ALL cell lines [69,70]. The antileukemic action of BRT deserves further studies.

## 5. Mechanism of Action

### 5.1. Interference with the NFκB Pathway

The nuclear factor-κB (NFκB) family of transcription factors is a master regulator of immune functions. The dysregulated activation of this pathway contributes to the pathogenesis of multiple diseases, including autoimmune and inflammatory diseases, and cancers [71,72,73]. BRT affects the NFκB pathway, notably via an induced up-regulation of proteins p50 and p65, and down-regulation of phospho-p65, as shown in PANC-1 pancreatic cancer cells [64]. Most likely, BRT reacts with Cys-38 residue of NFκB p65 subunit, as observed with other SLs. This NFκB pathway is frequently activated in pancreatic cancers [74], and the suppression of phospho-p65 contributes to the anti-inflammatory and antiproliferative action. Decreasing phospho-p65 is also a means to augment the efficacy of chemotherapy [75]. Inhibition of phospho-p65 protein expression by BRT has been confirmed using BEL 7402 and HepG2 hepatocellular carcinoma cells [63]. The BRT-induced modulation of the NFκB pathway is linked to immune response. In mice, BRT was found to increase the blood level of interleukin-2 (IL-2) acting as a T-cell growth factor (IL-2 can induce growth and differentiation of NK and B cells, CD4^+^ and CD8^+^ T cells), and to decrease the level of IL-10 which inhibits IL-2 production and inactivates CD4^+^ T cells. BRT seems to enhance the immune response, via the opposite regulation of IL-2 (up) and IL-10 (down), thereby inhibiting tumor escape and progression (Figure 4) [66]. The modulation of the NFκB pathway contributes to the anti-inflammatory action of BRT, notably via inhibition of different inflammatory mediators (nitric oxide, prostaglandin E2) and induction of anti-inflammatory signals (inducible NO synthase, cyclooxygenase-2) in lipopolysaccharide-stimulated macrophages [76,77]. This NFκB-dependent mechanism of BRT is common to several pseudoguaianolides, including helenalin, bigelovin and ergolide [78,79,80,81] and to dimeric sesquiterpene lactones, such as japonicone A [82]. However, it has also been shown that the biological effects of these SLs are not only due to NFκB inhibition but must be coupled to other mechanisms, such those described below [83].

### 5.2. Blockade of the Keap1-Nrf2 Pathway

**Figure 5:**
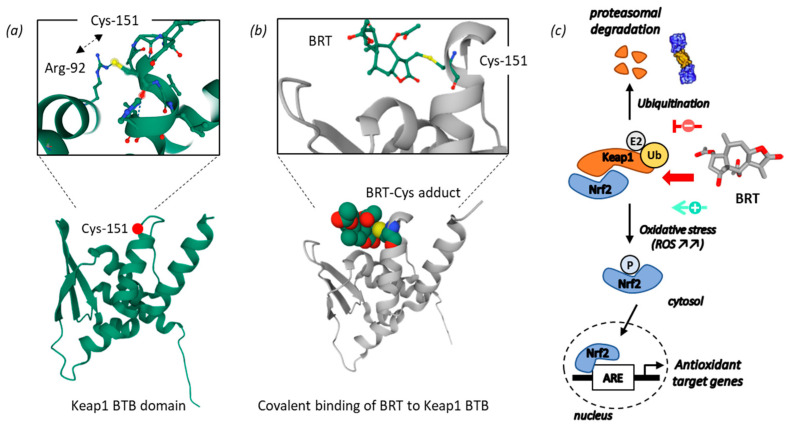
Modulation of the Keap1–Nrf2 pathway by BRT. (**a**) Molecular model of the BTB domain of Keap1 (PDB code: 4CXI) to show the position of residue Cys-151 interacting with Arg-92 to stabilize the protein. (**b**) Model of BRT covalently bound to the Cys-151 residue of Keap 1 (PDB code: 5GIT). (**c**) Nrf2–Keap1 signaling cascade blocked by BRT. Covalent binding of BRT to Cys-151 of Keap 1 leads to activation of Nrf2 (which thus escapes ubiquitination and proteasome-dependent degradation), phosphorylation and nuclear translocation, and then activation of the transcription of antioxidant target genes.

The mode of action of BRT is multifactorial. One of the prime pathways regulated by BRT is the Keap1-Nrf2 pathway which is the main protective response to oxidative and electrophilic stresses. BRT is a potent inducer of the transcription factor Nrf2 (nuclear factor erythroid 2-related factor 2) and does so via a direct binding to a conserved cysteine residue (Cys-151) of Keap1 (Kelch-like ECH-associated protein-1) (Figure 5). Keap1 is a homodimeric protein which orchestrates a complex transcriptional program in responses to a variety of oxidative stress conditions [84]. Under normal conditions, Keap1 traps Nrf2 in the cytoplasm and promotes its degradation by the 26S proteasome. Because of Keap1 binding, BRT inhibits Keap1-mediated ubiquitination of Nrf2 and triggers activation of Nrf2 [85]. Keap1 is part of an E3 ubiquitin ligase and normally targets Nrf2 for ubiquitination and proteasome-dependent degradation. Covalent modification of Keap1 at Cys-151 produces a conformational change in Keap1, which induces the dissociation of the Keap1–ubiquitin ligase complex [86,87]. Cys-151 is one of the three major cysteine sensors of Keap1 in stress response [88]. This cysteine residue, being particularly reactive [89], is exploited by diverse natural products to modulate the function of Keap1, such as curcumin [90,91], the alkaloid (+)-clausenamide [92], 4β-hydroxywithanolide E [93], artemisin derivatives [94] and other products [95,96,97,98]. A covalent binding of the pentacyclic oleanane triterpenoids to Cys-151 in the BTB domain of human Keap1 has been demonstrated. The crystal structures of the complex between Keap1 and the oleanic acid derivative CDDO [99] and TX64014 [100] have been fully characterized. A similar structure has been solved with BRT (Figure 4) [85]. This Cys-151 residue could be viewed as the Achilles’ heel of Keap1, targeted by BRT and other reactive molecules. This is the main molecular basis of the antioxidant action of BRT.

In the presence of BRT, NRF2 can escape ubiquitination. Then, it can accumulate within the cell and translocate to the nucleus, where it promotes its antioxidant transcription program. The Keap1–Nrf2 pathway is implicated in multiple human diseases, as a regulator of oxidative stress [101]. Nrf2 inhibitors are searched for because the protein is frequently aberrantly activated in cancer cells. Both inhibitors and activators are investigated: inhibitors for direct action on cancer cells, and inducers to provide a protective action, protecting from cell damages induced with conventional chemotherapeutic anticancer agents [102]. Induction of the Nrf2 protective pathway by BRT is interesting, not only to protect from chemotherapy-induced cell damages, but also in other pathologies, for example in the frame of tissue injury after cerebral ischemia [85].

### 5.3. Modulation of c-Myc/HIF-1α Signaling Axis

**Figure 6:**
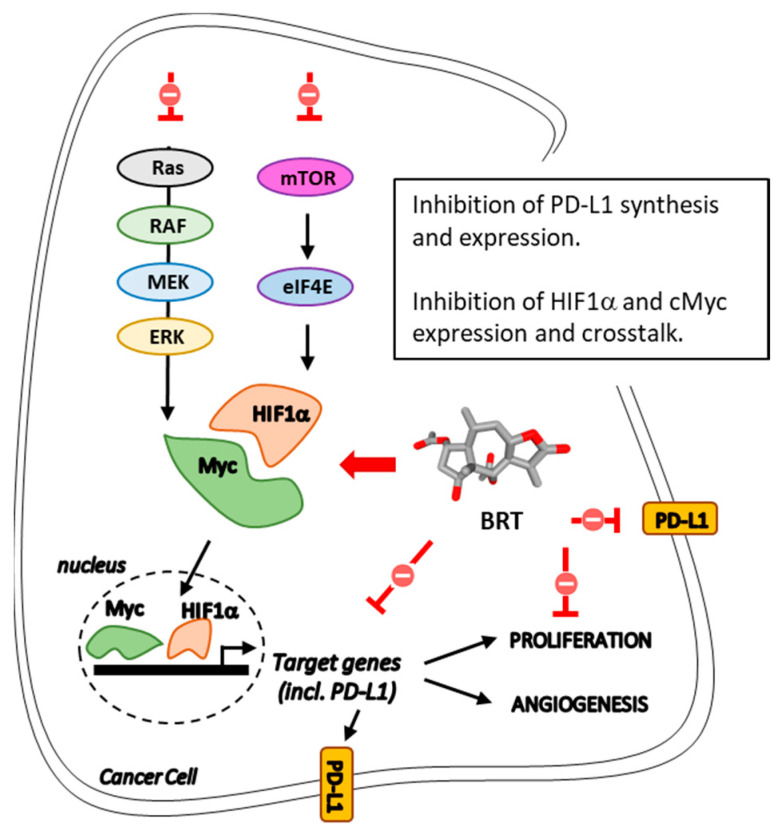
Modulation of the cMyc-HIF1α pathway by BRT. The compound downregulates expression of both cMyc and HIF1α, presumably via an interference with upstream effectors, such as mTOR and Rs/RAF. BRT also inhibits the direct interaction between cMyc and HIF1α. These different actions lead to a reduction in expression of specific proteins, including PD-L1, of which membrane expression is massively attenuated by BRT. These effects lead to inhibition of angiogenesis and cancer cell proliferation (adapted from [67]).

Beyond its antioxidant effect, BRT also functions via the regulation of cell cycle progression in cancer cells. In MOLT-4 cells, BRT was found to prevent the S-phase cell cycle transition through the up-regulation of proteins p21 (cip1) and p27 (kip1) which are the two main cyclin-dependent kinase (CDK) inhibitors [68]. In MCF-7 and MDA-MB-468 breast cancer cells, BRT was found to reduce expression of cyclin D1 and CDK4 proteins, leading to arrest of the cell cycle in the G1-phase [61]. In HCT116 colon cancer cells, BRT inhibited the expression of cyclin D1 and erythropoietin [67]. In this later case, the mode of action invoked is a blocking of the interaction between the hypoxia-inducible factor 1 alpha (HIF-1α) and c-Myc, which resulted in blocking the activation of their downstream targets. It is known that these two transcription factors, c-Myc and HIF-1α, cooperate to promote cancer cell growth and progression [103,104,105,106]. In fact, HIF-1α induces cell cycle arrest by functionally counteracting Myc [107]. At this level, the mode of action of BRT (Figure 6) is reminiscent of that of the quassinoid brusatol, which is also an inhibitor of Nrf2 and c-Myc, which increases HIF-1α degradation to induce cell death of cancer cells [108]. Similar effects have been reported with other natural products, such as triptolide [109]. The exact molecular target of BRT remains to be determined. Inhibition may occur through a direct targeting of HIF-1α or via an upstream target within the mTOR/4E-BP1 signaling pathway which controls HIF-1α protein synthesis [67]. A similar action has been invoked with the dietary monoterpene perillyl alcohol, for example [110].

In cells, energy metabolism is regulated by the activity of several transcription factors, among which is the triad of c-Myc, HIF-1 and p53, essential to control glycolysis [111]. The interaction between HIF-1α and c-Myc is key to the adaptation of cancer cells to the hypoxic microenvironment and the malignant progression [112,113]. The two proteins collaborate to enhance the cancer cell’s metabolic needs through increased uptake of glucose and its conversion to lactate [105]. BRT has the capacity to inhibit the expression of both HIF-1α and Myc, as well as the crosstalk between the two proteins. This effect contributes to shaping the immune response because these two key actors are known to coordinate T cell metabolic reprogramming [114].

BRT also negatively regulates downstream targets, such as the immune checkpoint protein PD-L1 (programmed cell death-ligand 1) that is frequently overexpressed in tumor cells. PD-L1 is largely implicated in the escape of tumor cells to T-cell killing, and contributes to promote tumor cell survival, migration and proliferation. BRT has been found to markedly inhibit the expression and protein synthesis of PD-L1 in various types of cancer cells, via HIF-1α/c-Myc [67]. Both PD-L1 and HIF-1α play major roles in tumor immune evasion. Notably, HIF-1α induces expression of a variety of immunosuppressive molecules and contributes to the regulation of PD-L1 expression on cancer cells [115]. The co-overexpression of PD-L1 and HIF-1α in tumor tissues, for example in hepatocellular carcinoma tissue, is associated with a high risk of recurrence or metastasis [116]. Thus, interfering with the HIF-1α/PD-L1 axis with BRT can be an efficient way to restore an immuno-sensitivity and to facilitate the action of cytotoxic (CD8^+^) T cells.

**Figure 7:**
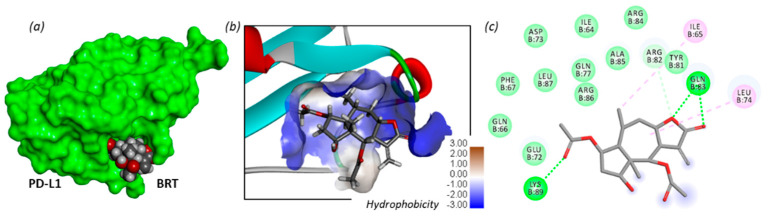
Molecular model of BRT bound to PD-L1 (PDB structure 5O4Y). (**a**) BRT binding to PD-L1 (in green). (**b**) A detailed view of a stick model of BRT binding to PD-L1, with the hydrophobic zone surrounding the biding site (color code indicated). (**c**) Binding map contacts for BRT bound to PD-L1 (green dashed lines correspond to H-bonds). The site is centered around residue Ile-64 of PD-L1. Calculated potential energy of interaction ΔE: −56.50 kcal/mol.

This is an important discovery, because PD-L1 is a co-inhibitory molecule often expressed on tumor cells and considered a prime target in oncology. PD-L1-targeted monoclonal antibodies are largely used to treat solid tumors. The use of small molecules regulating PD-L1 expression can be extremely useful to design chemo-immunotherapy combinations [117]. Zhang and co-workers have shown that BRT has the capacity to down-regulate PD-L1 expression in various cancer cell types (HeLa, Hep3B, HCT116, A549), and they suggested that the compound could directly bind to PD-L1, on the basis of a molecular docking analysis [67]. Our own in silico analysis has confirmed that the binding of BRT to monomeric PD-L1 is conceivable. BRT can bind to a cavity of PD-L1, centered on residue Ile-64, positioning its lactone unit toward residue Gln-83 and the carbonyl of the 2-acetyl group interacts with Lys-89 residue (Figure 7). It is unclear at present if the effect of BRT on PD-L1 is due to a direct effect (protein-binding) and/or an indirect effect, via the suppression of the crosstalk between HIF-1α and Myc by BRT. Nevertheless, the downregulation of PD-L1 by BRT is an essential contributor to the anticancer action of this sesquiterpene lactone.

### 5.4. Modulation of Other Signaling Pathways

BRT operates via multi-target and multi-pathway mechanisms. One of the signaling routes implicated in the mechanism of action of BRT is the AMP-activated protein kinase (AMPK) pathway. BRT has been found to activate AMPK in liver cancer cells and to induce apoptosis and autophagy. The process is associated with the production of reactive oxygen species (ROS) promoted by BRT in hepatic cancer cells [62]. Increased ROS generation is necessary to trigger activation of the mitochondrial apoptotic pathway by BRT in breast and pancreatic cancer cells [64,118]. The ROS-dependent mechanism is also central to the anticancer action of gaillardin and bigelovin [119,120,121]. In addition, the compound BRT modulates the AKT-FOXO1 signaling axis in human pancreatic cancer cells, decreasing the level of phospho-AKT and inducing nuclear accumulation of the transcription factor FOXO1 in AsPC-1 and Panc-1 pancreatic cancer cells [64].

These different mechanisms or pathways modulated by BRT are interconnected. ROS-modulating drugs can effectively regulate PD-L1 expression on cancer cells [122] and NFκB regulates PD-L1 expression in cancer [123,124]. The transcription factor NFĸB functions as an essential regulator of the immune response, and cell proliferation and transformation [125]. Other natural products are capable of down-regulating PD-L1 via inhibition of NFĸB, such as ginsenoside Rk1, oleanic acid, hesperidin and sesamin [126,127,128,129]. The NFĸB and PD-1/PD-L1 axes provide a link between inflammation and cancer. Obviously, BRT targets multiple pathways and the combined action contributes to its marked antitumor action.

## 6. Thiol Reactivity

**Figure 8:**
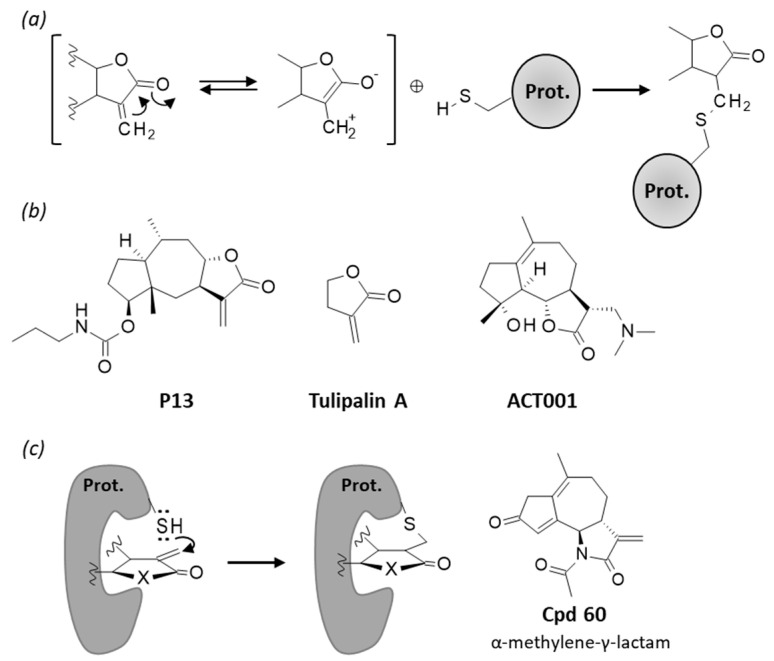
Thiol reactivity of BRT and related SLs. (**a**) Michael reaction between the highly reactive α-methylene-γ-lactone unit and an exposed sulfhydryl group of a protein to form a covalent drug–protein adduct (adapted from [4]). (**b**) Structures of compounds tulipalin A, P13 and ACT0001. (**c**) Structure of the reactive α-methylene-γ-lactam compound 60, acting as a covalent inhibitor of NFκB. The compound has been shown to form stable adducts with cysteamine [145].

The α-methylene-γ-lactone moiety of these molecules is a reactive entity, capable of Michael-type addition with biological nucleophiles, in particular with the sulfhydryl groups of proteins [4] (Figure 8). BRT has been shown to bind covalently to a cysteine residue (Cys-151) of Keap1 [85], and helenalin shows a high reactivity toward specific thiol-containing proteins, such as the p65 subunit of NFκB [10,11]. Helenalin potently inhibits human telomerase, an enzyme activated in most cancer cells [130,131]. The inhibition has been attributed to alkylation of cystein-445 residue of telomerase [132]. A pulchellin derivative designated P13 (2-desoxy-4β-propylcarbamate-pulchellin, Figure 8) was found to bind covalently to cytsein-452 of Janus kinase 2 (JAK2), a key kinase implicated in STAT3 signaling [133]. The same mechanism of JAK2 covalent inactivation has been advanced for bigelovin [134]. The pseudoguaianolide 2-desoxy-4-epi-pulchellin, isolated from *Carpesium faberi* and *C. abrotanoides*, also presents a reactive group and functions as an anticancer STAT3 inhibitor [135]. The anticancer activity of other types of sesquiterpene lactones equipped with an α-methylene-γ-lactone group has been shown to rely on their thiol reactivity. This is the case, for examples, for vernolide-A and vernodaline from *Vernonia* species [136] and parthenolide from *Tanacetum parthenium* [137,138]. This latter compound, parthenolide, has the ability to modify the redox state of critical exposed (exofacial) thiol groups of proteins [139]. Therefore, the bioactivity of SL compounds cannot be attributed to the modification of a unique target. Their mechanism of action has been qualified as being polytargeted, leading to a multidirectional activity [5]. This multimodal action confers potent anticancer activities to helenalin, but also enhances the risk of unwanted toxicity.

Helenalin can cause allergic reactions [140,141]. The allergenic potential of these sesquiterpene lactones is a concern, strongly limiting their development as drugs to treat human diseases [142,143,144]. However, chemical efforts are deployed to reduce this limiting factor. New compounds with the exomethylene unit replaced with a non-reactive group have been made. Some of these compounds, more water-soluble than natural SLs, have been found to maintain a high level of STAT3 inhibition and marked cytotoxic effects toward cancer cells [135]. Another option to tune the electrophilicity of the α-methylene-γ-lactone is to replace this unit with a less reactive α-methylene-γ-lactam (Figure 8), as performed recently with parthenolide derivatives with the objective to mute the nonspecific thiol reactivities. In this case, the chemical nature of the group on the lactam nitrogen greatly impacted the reactivity of the α-methylene–γ-lactams toward a model thiol compound (cysteamine). α-Methylene–γ-lactam guaianolides maintaining an inhibitory activity toward NFκB were identified, but there is positive correlation between the thiol reactivity of the compounds and their NFκB inhibitory activity [145]. Another option consists of removing the exomethylene unit, replacing it with a less- or non-reactive group. This is the case for the parthenolide derivative ACT001 (Figure 8). This water-soluble compound (dimethylaminomicheliolide) exhibits a higher plasma stability than parthenolide and was found to target glioma stem-like cells through regulation of the protein AEBP1 (adipocyte enhancer binding protein 1) [146]. It has revealed interesting anti-neuroinflammatory properties, attenuating microglial activation in a mouse model of Parkinson’s disease [147]. It is also an NFĸB inhibitor, potentially useful for the treatment of idiopathic pulmonary fibrosis [148]. The same type of modifications of the exomethylene unit could be applied to the BRT skeleton.

## 7. Discussion

**Figure 9:**
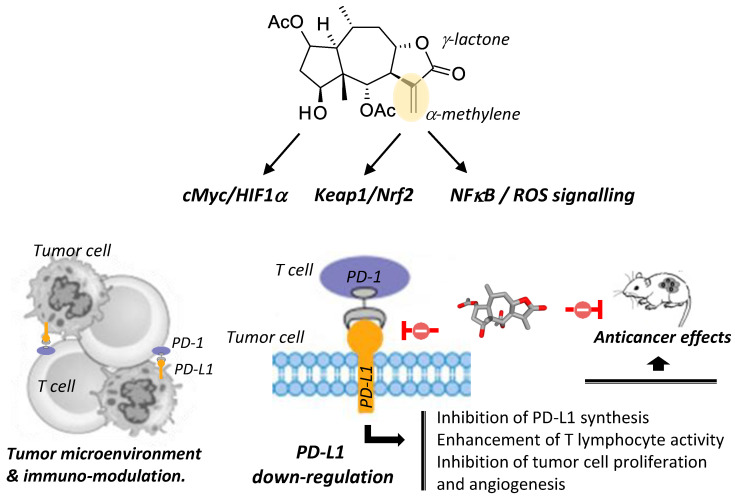
A schematic illustration of the anticancer mechanism of BRT. The reactive α-methylene unit of the compound plays a major role in inhibition of NFκB and activation of Keap1/Nrf2 signaling. In addition, the compound induces a down-regulation of the membrane ligand PD-L1 expressed on cancer cells, thereby blocking the PD-1/PD-L1 immune checkpoint and activating cytotoxic T lymphocytes. Down-regulation of PD-L1 by BRT can occur directly via binding to PD-L1 and indirectly via repression of the cMyc/HIF1α signaling route. Collectively, the effects lead to inhibition of cancer cell proliferation and angiogenesis.

BRT is one of the many bioactive natural products, with antioxidant, anti-inflammatory and anticancer properties isolated from *Inula* species. More than 100 compounds have been isolated thus far [20,149,150]. BRT is certainly one of the most active compounds found in *I. britannica* L. There are important structural and functional similarities among pseudoguaianolide-type sesquiterpenes. BRT is structurally close to ergolide, bigelovin and helenalin, and all these compounds present marked antitumor activities. These different compounds all modulate the NFĸB pathway and trigger ROS-mediated apoptosis of cancer cells. However, there are also mechanistic differences between these natural products. BRT targets (i) the NFĸB-ROS pathway, (ii) the Keap1-Nrf2 pathway, (iii) the HIF1α/PD-L1 pathway and possibly other components of the cell machinery, such as AMPK (Figure 9). Recently, helenalin was found to target thioredoxin reductase-1 (TrxR1) in human prostate cancer cells, suppressing TrxR1 expression in these cells [151]. This mechanism could well be utilized also by other pseudoguaianolides such as BRT, in parallel to other mechanisms. There are perhaps too many mechanisms for these reactive compounds. The multiplicity of points of action, the so-called multitargeted activity, can be beneficial to modulate the activity of difficult-to-drug target proteins (or unexplored targets), and to combat aggressive cancers with generally multiple altered signaling pathways. However, it can also be a potential source of unwanted toxicities, especially with highly reactive compounds such as SLs. The α-methylene-γ-lactone moiety of these compounds can lead to non-specific thiol reactivities with a variety of proteins. The α-methylene-γ-lactone is a photochemical group possibly implicated, if not directly responsible for, skin damages induced by some SLs compounds, such as chronic actinic dermatitis [152]. The minimal α-methylene-γ-lactone unit, corresponding in fact to the natural product tulipalin A (Figure 8), is a well-known allergen with immunotoxic properties [153]. As mentioned above, there are options to reduce or to abolish this non-specific reactivity, via the modification/suppression of α-methylene-γ-lactone unit.

The immuno-modulatory capacity of BRT is a new and key element of its mechanism of action, which heightens the interest of the compound. Modulation of the tumor microenvironment is an essential component of a global antitumor action. BRT stabilizes T cells and enhances their ability to kill tumor cells [67]. At first sight, the ability of BRT to promote T-cell functions could be a little surprising because a few years ago, the related product helenalin has been shown to suppress immune functions of activated CD4^+^ T cells [154]. However, it makes sense when considering that the related compound ACT001 (without an α-methylene) can also reduce PD-L1 expression in cancer cells [155]. ACT001 downregulates PD-L1 expression in glioblastoma cells through inhibition of the phosphorylation of the transcriptional regulator STAT3, leading to the inhibition of PD-L1 transcription [155]. The invoked mechanism is distinct from that reported with BRT, which inhibits PD-L1 expression through the blockade of the HIF1α–Myc interaction, and possibly via a direct binding to PD-L1 as well [67]. Whatever the exact molecular mechanism, the two compounds induce a similar down-regulation of PD-L1 expressed on cancer cells and enhance the activity of cytotoxic T lymphocytes. This property confers to the compounds the ability to modulate the tumor microenvironment.

## 8. Conclusions

BRT is a sesquiterpene lactones known for more than fifty years, initially considered as an anti-inflammatory and antioxidant agent and later characterized as an anticancer compound. It is a complex, reactive molecule susceptible to forming adducts with free thiol group in proteins, but this non-specific reactivity can be (and probably should be) controlled upon removal or modification of the exomethylene unit of BRT. The compound reduces cancer cells proliferation, survival and migration through multiple signaling pathways, notably via modulation of the NFκB, Keap1/Nrf2 and HIF1α pathways. In addition, an immunomodulatory function of BRT has been discovered recently. BRT functions as a PD-L1 inhibitor, acting directly and/or indirectly on PD-L1, to reduce its expression, and thereby inhibiting cancer cell proliferation and angiogenesis (Figure 9) [67]. This discovery calls for the analysis of other similar SLs as potential PD-L1 regulators. The time has come to reconsider the use of sesquiterpene lactones for the treatment of cancer, taking into account their under-studied immunomodulatory functions, in addition to their well-established antioxidant, anti-inflammatory and anti-proliferative effects. The capacity of BRT to regulate T cell activity calls for a deeper investigation into the capacity of (exomethylene-modified) SLs to remodel the tumor microenvironment and promote an effective anticancer immune response.
